# Pickering Emulsions Stabilized by Conjugated Zein-Soybean Polysaccharides Nanoparticles: Fabrication, Characterization and Functional Performance

**DOI:** 10.3390/polym15234474

**Published:** 2023-11-21

**Authors:** Lili Yao, Ying Wang, Yangyang He, Ping Wei, Chen Li, Xiong Xiong

**Affiliations:** 1 College of Food Science and Light Industry, Nanjing Tech University, Nanjing 211816, China; llynjut@126.com (L.Y.); 202261218186@njtech.edu.cn (Y.W.); heyangyang0928@163.com (Y.H.); lichenfs@njtech.edu.cn (C.L.); 2 College of Biotechnology and Pharmaceutical Engineering, Nanjing Tech University, Nanjing 211816, China; weiping@njtech.edu.cn

**Keywords:** zein, soybean polysaccharide, Maillard reaction, nanoparticles

## Abstract

This study aims to fabricate zein-based colloidal nanoparticles, which were used to stabilize Pickering emulsions, by conjugation with soybean polysaccharide (SSPS) through the Maillard reaction. The physicochemical properties of the conjugated particles as well as the physical and oxidative stability of the fabricated Pickering emulsion that utilized conjugated colloidal particles with the volumetric ratio of water and oil at 50:50 were investigated. The grafting degree of zein and SSPS was verified through examination of FT-IR and fluorescence. Moreover, the conjugated Zein/SSPS nanoparticles (ZSP) that were prepared after dry heating for 48–72 h exhibit excellent colloidal stability across a range of pH values (4.0–10.0). Further, the wettability of ZSP decreased based on a contact angle analysis of θ~87°. Confocal laser scanning microscopy (CLSM) images indicated that ZSP particles were located around the oil droplets. Additionally, the ZSP effectively improved the oxidative stability of the Pickering emulsions, as demonstrated by a significant decrease in both peroxide value (PV) and thiobarbituric acid reactive substances (TBARS). The results of this study demonstrate that ZSP represents a promising food-grade Pickering emulsifier, capable of not only stabilizing emulsions but also inhibiting their oil oxidation.

## 1. Introduction

In recent decades, the preparation of Pickering emulsions using biopolymer nanoparticles as stabilizers has become the focus of research because of their great potential to improve food textures, reduce lipid oxidation and deliver some bioactive compounds [1,2]. Many biodegradable materials, including proteins, starch, cellulose and chitosan and/or their poly-complexes, have been fabricated into colloidal particles as Pickering stabilizers [3,4]. Among the above natural polymers, protein-based particles are particularly attractive candidates because they are amphiphilic macromolecules composed of amino acids that can simultaneously anchor at the oil-water interface and maintain emulsion stability through highly viscoelastic films and spatial site resistance [5]. In addition, the advantages of proteins as stabilizers also include their sustainability, biosafety, adjustable three-dimensional structures and acceptable food composition properties.

Zein is a natural water-insoluble protein that contains over 50% of non-polar amino acids in its molecular structure [6]. Due to the difference in solubility of zein in water and aqueous ethanol solutions (55–90%), zein nanoparticles (ZP) obtained easily by the anti-solvent method can be used to prepare a non-surfactant O/W Pickering emulsion with a size distribution of 10~200 nm [7]. Hence, zein has become one of the most widely utilized protein-based Pickering emulsion stabilizers. For instance, Xu et al. [8] proposed the stabilization of Pickering emulsions of clove essential oil using ZP as the sole stabilizer that showed smaller droplet sizes and more uniform distribution. However, it was also observed that the stratification and demulsification of the emulsions occurred at the early stage of storage. This was related to the aggregation caused by the inherent hydrophobicity of ZP, leaving oil droplets partially unwrapped [9]. Furthermore, ZP were unstable against coalescence and aggregation under some conditions such as high temperatures, isoelectric pH and high ionic strength [6]. Therefore, it is necessary to enhance the stability of zein colloidal particles via modification or combination with other biopolymers and expand their application in Pickering emulsions.

It is generally accepted that the conjugation of protein polysaccharides by means of the Maillard reaction (MR) is a promising approach to improve the physicochemical and functional properties of protein [10]. The MR involves non-enzymatic reactions among the free amino acids of the protein and the carbonyl groups of reducing sugars via the covalent bond, which can irreversibly alter protein structures and ultimately modify their functionalities including solubility, physical stability, antioxidant capacity and emulsification performance [11]. It is worth mentioning that glycosylated protein demonstrates greater stability across diverse environments and possesses greater tolerance to fluctuations in pH, temperature and ionic strength when compared to non-glycosylated protein [12]. So far, a number of studies have found that the creation of stable protein nanoparticles can be achieved through the MR, including enhancing the thermal stability of Whey protein isolate by conjugation with dextran [13], increasing the physical and oxidative stability of sodium caseinate by conjugation with gum Arabic [14], and improving the delivery performance of zein by conjugation with chitosan oligosaccharide [15]. Accordingly, it is speculated that conjugated protein could be used for the fabrication of low environment-sensitive nanoparticles instead of pure proteins.

Soybean polysaccharides (SSPS) are a kind of water-soluble polysaccharide with a pectin-like structure. This acidic polysaccharide is composed of a highly branched rhamnogalacturonan backbone and homogalacturonan, which is linked to protein moiety via covalent bonds [16]. Numerous proteins, including sodium casein [17], soybean protein isolate [18] and soy glycinin [19], have been conjugated with SSPS using the MR to enhance their emulsification and thermal stability. Based on these attributes, we believe that it is of great significance to investigate the physical stability of Zein/SSPS-conjugated nanoparticles (ZSP) in the context of Pickering emulsion construction.

Hence, the objective of the present study is to investigate the preparation and characterization of an emulsion stabilized by ZSP at different MR conditions. The impact of thermal treatments on the structural and physicochemical characteristics of ZSP is analyzed through the degree of grafting, particle size, zeta potential, Fourier transform infrared (FTIR) spectroscopy, fluorescence spectroscopy, sodium dodecyl sulfate-polyacrylamide gel electrophoresis (SDS-PAGE) and contact angle measurements. Afterwards, the creaming index (CI), droplet diameters and physical and oxidative stability of the Pickering emulsions containing flaxseed oil are also characterized.

## 2. Materials and Methods

### 2.1. Materials

Zein was purchased from Sigma Aldrich (St. Louis, MO, USA). Soybean polysaccharide (purity 85.1%) was obtained from Fujian Quanzhou Weibo Food Co., Ltd. (Quanzhou, China). Ortho-phthalaldehyde (OPA), 1,1,3,3-tetraethoxypropane and cumene hydroperoxide were obtained from Macklin Biochemical Co., Ltd. (Shanghai, China). Flaxseed oil without added antioxidants was purchased from a supermarket in Nanjing. Other chemicals and reagents were of analytical grade. The sample solutions were prepared using deionized water (Milli-Q Academic, Beijing, China).

### 2.2. Preparation of Zein/SSPS Conjugates

Zein (20 g/L) and SSPS (20 g/L) were separately dispersed in Milli-Q water (pH = 6.2) and magnetically stirred for 12 h. The pH of the solution was adjusted to either pH 7.0 or 10.0 with 1 M NaOH. Then, Zein/SSPS dispersions were selected at mass ratio 1:1 and were dehydrated by freeze drying for 48 h. After freeze drying, the Maillard reaction of the resulting Zein/SSPS mixture was promoted by incubating the powder in a desiccator pre-heated at 80 °C and 79% relative humidity for different times (24, 48, 72 h). The Zein/SSPS conjugates (ZSC) of different conjugation degrees were stored in a dark and dry place for further characterization.

### 2.3. Preparation of Zein/SSPS Conjugated Nanoparticles (ZSP)

Zein/SSPS-conjugated nanoparticles were prepared using the anti-solvent precipitation method. Briefly, a certain amount of ZSC powder was dissolved in an 85% (*v*/*v*) aqueous ethanol solution under magnetic stirring for 30 min, and then sample solutions were centrifuged at 4000 rpm for 30 min to remove most of the unreacted SSPS. Afterward, the supernatant was added drop-wise into 120 mL of deionized water, and the resulting mixture was concentrated to 40 mL using a rotary evaporator (45 °C). The final concentration of ZSP in the particle dispersions was 2% (*w*/*v*), which was then adjusted to a pH of 4.0 with 1 M HCl. Zein nanoparticles (ZP) were prepared using the same procedure as that of the control. A part of the resulting nanoparticle dispersion was centrifuged at 9000 rpm for 10 min to collect the insoluble portion, which was subsequently rinsed in deionized water and then lyophilized and milled into powders. The sample information of five different zein-based particles are summarized in Table 1.

### 2.4. Characterization of Zein/SSPS Conjugated Nanoparticles (ZSP)

#### 2.4.1. Determination of Free Amine Content and Degree of Grafting (DG)

The free amine contents of the ZSP were characterized according to a previous report with slight modifications [20]. OPA of 40 mg was dissolved in 1 mL of ethanol, 2.5 mL of 20% (*w*/*w*) SDS and 25 mL of 0.1 mol/L borax, after which 0.1 mL of 2-mercaptoethanol was added and dilution to 50 mL was finally undertaken with distilled water. Then, 0.2 mL of the sample solution was mixed with 4 mL of the OPA reagent and incubated at 35 °C for 2 min. Absorbance was measured at 340 nm by a UV–Vis spectrophotometer (UV-1900, Shimadzu, Kyoto, Japan). Leucine was used as the standard to calculate the content of the free amino groups. The formula used was as follows:

$$DG\left(\%\right)\;=\;\frac{A_{0}-A_{t}}{A_{0}}\times 100\%$$

where *A*_0_ and *A_t_* are the free amine content of native zein and Zein/SSPS conjugates, respectively. 

#### 2.4.2. SDS-PAGE

The SDS-PAGE was used to monitor changes in molecular weight (MW) in the Zein/SSPS conjugates at different incubation times according to the methodology by Laemmli [21]. This experiment was performed in a 0.5 mol/L Tris-HCl buffer (pH 6.8), in which concentrations of the stacking gel and separating gel were 5% and 10%, respectively. Specifically, the sample solution (1 mg/mL, 80% ethanol solution) was diluted with an equal volume of Tris-HCl buffer solution (0.5 M, pH 6.8) and vortexed and heated to 100 °C for 5 min. Then, 15 μL of this suspension for each treatment was loaded into each gel well, followed by electrophoresis at a constant voltage (120 V for 45 min) in a Tris-glycine-SDS buffer (pH 8.8) containing 0.5% SDS (*w*/*v*). Standard proteins were used as a marker (14.4–270 kDa). After migration, the gels were stained using Coomassie Brilliant Blue R-250 for 2 h and destained with an acetic acid-decolored solution to visualize the protein.

#### 2.4.3. FTIR Analysis

The sample powder was mixed with KBr at a certain proportion of 1:100 (*w*/*w*) and then pressed into disks. The FTIR spectra was determined using a Nicolet 380 spectrometer (Thermo Fisher Scientific, Waltham, MA, USA). Spectrograms of the sample were collected directly in the range 400–4000 cm^−1^ at a resolution of 4 cm^−1^ using 32 scans. 

#### 2.4.4. Fluorescence Spectroscopy

The sample was dissolved in 80% (*v*/*v*) ethanol at a protein concentration of 0.2 mg/mL. A fluorescence spectrometer (F-4700, Hitachi, Tokyo, Japan) was used for scanning. The experimental parameters were set for endogenous fluorescence: the excitation wavelength was 280 nm, and scanning range was 290–450 nm. The widths of emission and excitation slit were fitted at 5 nm.

#### 2.4.5. Evaluation of Nanoparticle Stability

The freshly prepared conjugated nanoparticle suspension was adjusted to different pH values (4.0–10.0) to assess the pH stability. The size and zeta potentials of prepared samples were determined via dynamic light scattering (DLS) utilizing the Zeta sizer Nano-ZS (Malvern Instruments Ltd., Malvern, UK). The samples were diluted 20 folds with distilled water and then measured at 25 °C with the refractive index at 1.330.

#### 2.4.6. Wettability Measurements

The lyophilized ZP and ZSP samples were pressed into cylindrical tablets (13 mm × 2 mm), and an optical contact angle meter (LSA 100, Lauda Scientific, Lauda-Königshofen, Germany) was used to measure the oil-water contact angle (θo/w). A tablet was then immersed in corn oil and loaded onto a glass substrate. Then, a drop of water (2 μL) was slowly excluded, and a high-precision syringe was used to quickly place the particles on the surface of the tablet. A camera was used to photograph the shape of the water, and the θo/w via simulation was calculated using the Laplace-Young equation.

### 2.5. Preparation of ZSP-Stabilized Pickering Emulsions

A Pickering emulsion was formed by the ZSP dispersion and flaxseed oil. Briefly, the ZP-, ZSP_A_- and ZSP_B_-conjugated nanoparticles (protein concentration: 2 mg/mL) were mixed with flaxseed oil (1:1, *v*/*v*). A high-speed homogenizer T25 (14,000 r/min, 3 min, IKA Works, Wilmington, NC, USA) was used for the Pickering emulsions.

### 2.6. Emulsions Stability

The emulsions storage stability was investigated in accordance with the published method [22]. All samples were kept in the dark at 4 °C. The Pickering emulsion images were captured immediately and 15 days after synthesis. The creaming index (CI) was calculated based on the method [22] that uses the CI equation:

$$CI\left(\%\right)\;=\;\frac{H_{s}}{H_{t}}\times 100\%$$

where *H_s_* is the height of the emulsified layer, and *H_t_* is the total height of the emulsions.

### 2.7. Microstructure of Emulsions

All the Pickering emulsions were observed with an inverted fluorescence digital optical microscope (Leica, Germany). The images were taken 0 and 15 days after emulsification. Each emulsion was diluted with deionized water to the appropriate concentration, and the diluted emulsion was coated on a concave microscope slide for observation.

Emulsion droplets stabilized by Zein/SSPS-conjugated nanoparticles were observed using confocal laser scanning microscopy (ZEISS Inc., Jena, Germany). Samples were stained by a blended fluorescent dye solution (i.e., Nile Blue and Nile Red). CLSM images were obtained by selecting excitation at 488 nm (for Nile Red) and/or at 633 nm (for Nile Blue).

### 2.8. Oxidation Stability of Pickering Emulsions

Immediately after formation, the emulsions (20 mL) were placed in glass tubes with loosely sealed caps and kept in an oven for 15 days at 45 °C to accelerate the oxidation rate. The primary lipid oxidation product was measured via the peroxide value method (PV) according to Kargar and Spyropoulos’ method [23]. The PV of the emulsions were calculated using an external standard curve made of cumene hydroperoxide. In addition, the secondary oxidation products were determined using thiobarbituric acid reactive substances (TBARS) as described by Wang et al. [3]. The standard curve of 1,1,3,3-tetraethoxypropane was used to determine the concentration of TBARS. 

### 2.9. Statistical Analysis

All assays were carried out three times, and the data analysis was performed using the software SPSS Statistics 17.0 (SPSS Inc., Chicago, IL, USA). All results were recorded as mean ± standard deviation (SD). The comparison of mean values was undertaken to determine significant difference using a one-way analysis of variance (ANOVA) through a Duncan test at 95% confidence level (*p* < 0.05).

## 3. Results and Discussion

### 3.1. Characterization of Zein/SSPS Conjugated Nanoparticles (ZSP)

#### 3.1.1. Measurements of Free Amine Content and Degree of Grafting (DG)

**Figure 1:**
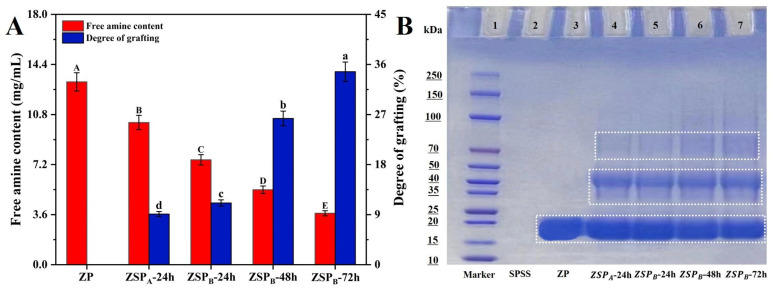
(**A**) Free amine content and DG of ZP and ZSP. (**B**) SDS-PAGE patterns of the zein nanoparticles (ZP) and Zein/SSPS conjugates nanoparticles (ZSP). (**A**) Different letters (A, B, C, D, E, a, b, c, d) indicate significant differences at *p* < 0.05. (**B**) Different numbers (1, 2, 3, 4, 5, 6, 7) in each lane represent Marker, SPSS, ZP, ZSP_A_-24 h, ZSP_B_-24 h, ZSP_B_-48 h, ZSP_B_-72 h.

The free amine content and degree of grafting (DG) are important parameters to characterize the extent of conjugation between protein and polysaccharide due to the Maillard reaction. Therefore, in order to estimate the extent of the Maillard reaction in the Zein/SSPS-conjugated nanoparticles (ZSP) designed in this study, we compared them to the non-conjugated corn protein nanoparticles (ZP) as a reference and tested the two parameters mentioned above (Figure 1A). The conjugate particles formed with SSPS at pH 7.0 and 24 h (ZSP_A_-24 h) served as another control. Compared to pure ZP, the free amine contents of ZSP decreased significantly (*p* < 0.05), indicating the successful conjugation between zein and SSPS that diminished the amino content of the protein [24]. The DG increased with the increasing of the pH of the Zein/SSPS system from pH 7.0 to pH 10.0 (Figure 1A). This behavior can be anticipated, considering that the introduction of active amino acids into carbonyl groups can be more pronounced through the further unfolding of the protein structure in a higher alkaline environment [25]. In addition, the DG of glycated ZSP_B_ significantly increased up to 34.7% with the increase of incubation time from 24 h to 72 h. The incubation time is also believed to affect the extent and rate of conjugation due to structural and conformational changes [26]. Zein, which is made up of typically dense spherical proteins, may need more time to unfold. Similarly, Cermeño et al. [27] also found that a longer incubation time led to a higher degree of glycation between whey protein isolate and carrageenan through dry heating.

#### 3.1.2. SDS-PAGE

SDS-PAGE was used to illustrate the covalent binding of zein to SSPS. As shown in Figure 1B, the main band of control, ZP (Lane 3), corresponded to the position of 20–25 kDa, which is a typical feature of α-zein [28], whereas SSPS (Lane 2) showed no distinct protein band [29]. After conjugation with the polysaccharide, the α-zein bands in ZSP_A_ (Lane 4) and ZSP_B_ (Lane 5–7) became less intense, particularly in ZSP_B_ where the intensity gradually decreased at the reaction times of 48 and 72 h. New bands near the loading ends of ZSP_A_ and ZSP_B_ occurred at higher positions than that of ZP. Moreover, the newly emerged bands were relatively wide and diffuse, indicating the production of a large number of high MW conjugates [30]. These compounds are produced by protein glycosylation and move more slowly through the gel. This finding is consistent with previous studies that showed that protein modification with SSPS delayed the rate of protein migration in SDS-PAGE, indicating the formation of covalent bonding between the two compounds [17,31].

### 3.2. Structural Properties of Zein/SSPS Conjugated Nanoparticles

#### 3.2.1. FTIR 

**Figure 2:**
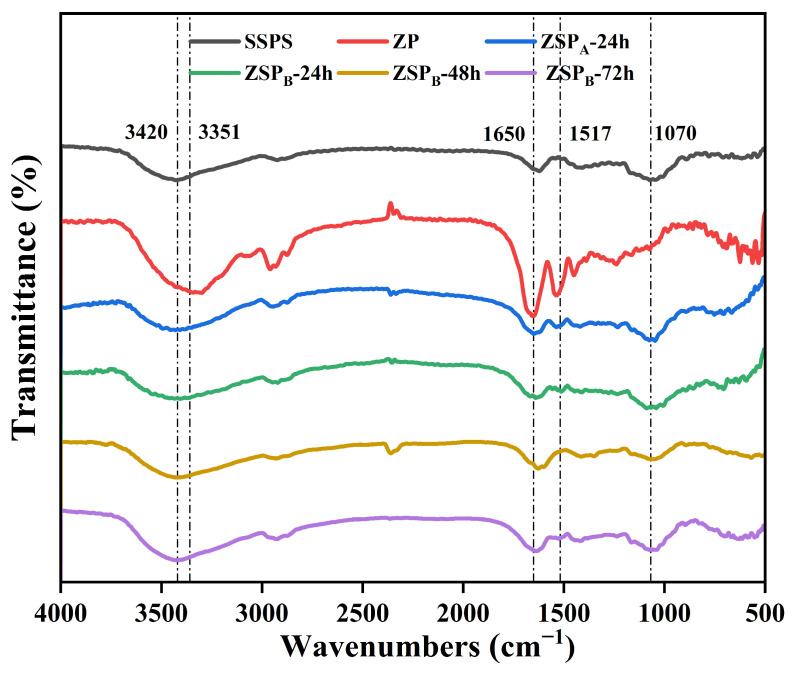
FTIR spectroscopy of ZP, SSPS and ZSP.

FTIR spectroscopy is an effective method to analyze protein-polysaccharide interactions and structural changes at the molecular level. The FTIR spectra of ZP, SSPS and ZSP with different incubation times are shown in Figure 2. Both pure zein and pure SSPS had broad absorption bands near 3420 cm^−1^, which can be associated with the stretching vibration of the O–H. The bands around 1650 and 1531 cm^−1^ in the spectrum of zein belong to amide I (C=O stretching) and amide II (N–H bend and C–N stretch), respectively, which are the unique spectral characteristic of proteins. The characteristic bands of SSPS were observed at 1070 cm^−1^, which indicates the presence of rhamnogalacturonan, whereas the one at 878 cm^−1^ is ascribed to β-glycoside linkages. These absorption bands are consistent with that of SSPS previously reported by other authors [29,32]. When zein was conjugated with SSPS, the ZSP_B_ differed from the zein in the range of 900–1650 cm^−1^, and a significant increase at 1070 cm^−1^ indicates that the degree of grafting increased and the glycated zein contained more C-OH than the original as the reaction time increased. This suggests that the SSPS covalently conjugated to the zein via the MR, which is in agreement with the result of the DG analysis. In addition, the amide I bands’ intensity decreased along with a slight shift in wavenumber from 1650 in zein to 1635 cm^−1^ in the conjugates (ZSP_B_-72 h) [33]. Abdelhedi et al. [34] also observed an upsurge in the covalent bonding between gelatin and sugar, which led to a change in amide I. Moreover, the intensity of Zein-SSPS conjugates decreased significantly compared to zein at amide II bands as the reaction duration increased (from 24 h to 72 h), suggesting the consumption of free amino groups and the formation of covalent bonds during the Maillard reaction. The FTIR data present persuasive evidence in support of the covalent linkage between zein and SSPS.

#### 3.2.2. Fluorescence Spectrum

**Figure 3:**
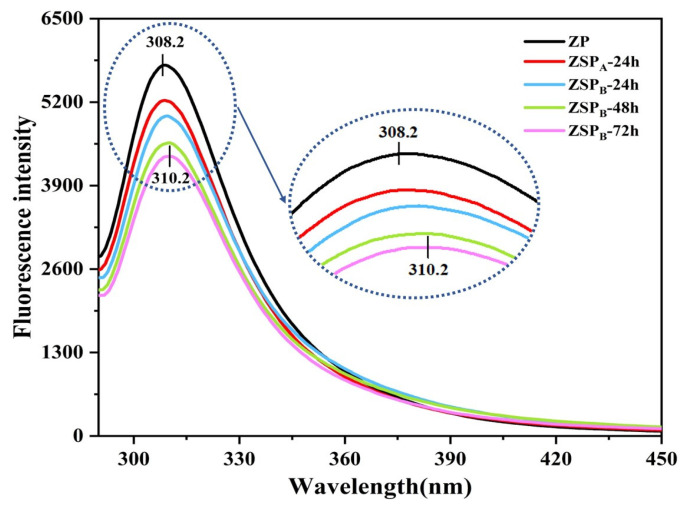
Fluorescence spectroscopy of ZP and ZSP at a concentration of 0.2 mg/mL.

The fluorescence spectrum is an effective detector of the tertiary structural changes of proteins. As shown in Figure 3, the zein molecules exhibited a maximum fluorescence intensity of around 308.2 nm. This may be due to the high concentration of tyrosine residues (about 5.1% *w*/*w*) in zein, while the content of tryptophan residues is negligible [35]. Huang et al. [36] reported that native zein dissolved in 75% ethanol and had maximum intensity at 309 nm. For the conjugated nanoparticles, the fluorescence intensity was appreciably reduced compared to that of the untreated zein, with a slight red shift appearing in the spectra, especially that of ZSP_B_-72 h (2 nm, from 308.2 nm to 310.2 nm). The red shift of the fluorescence peak indicates that the fluorescence emission group tyrosine is more exposed to the solvent, which means that the polarity of the microenvironment where the fluorescence emitting group is located increases [37]. This phenomenon indicates that as the degree of grafting increases, the tertiary structure becomes less compact, which enhances spatial stability and prevents protein aggregation during colloidal nanoparticle formation [38]. Additionally, it was observed that the fluorescence intensity decreased as the reaction time increased. This suggests that the Maillard reaction interrupted the conformational contacts within zein. As supported by Sheng et al. [39], a grafted polysaccharide can adopt a flexible random coil structure, which exerts a significant steric hindrance effect. This effect blocks the fluorescence signal of tyrosine residues, leading to a decrease in fluorescence intensity. It can also be said that with a high level of DG value, SSPS tends to form a molecular layer around zein, which reduces the detectable spontaneous fluorescence value. The findings of this study are in accordance with the previously reported fluorescence spectra of soy protein isolate-pectin conjugates [40].

### 3.3. Physical Stability of Native ZP and ZSP

#### 3.3.1. Effect of pH on the Stability of Native ZP and ZSP

The stability of protein-based nanoparticles is known to be highly sensitive under extreme pH conditions, especially extensive particle aggregation and sedimentation occurring at a pH close to pI. For this reason, we tested the stability of the conjugated reacting nanoparticles for 24 h (ZSP_A_-24 h, ZSP_B_-24 h) in the pH range of 4.0–10.0.

**Figure 4:**
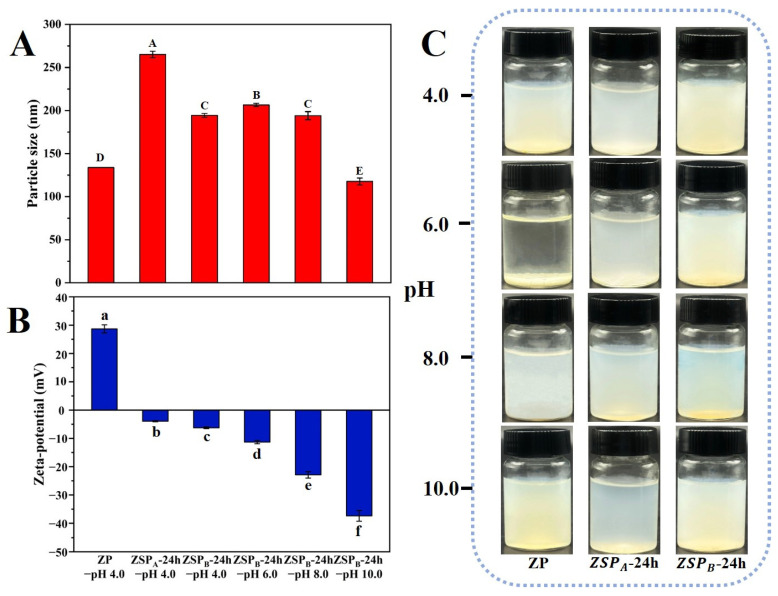
(**A**) Particle size, (**B**) zeta-potential and (**C**) a photograph of vials containing zein-based nanoparticles dispersions at different pH values (protein concentration: 2 mg/mL). Different letters (A, B, C, D, E, a, b, c, d, e, f) indicate significant differences at *p* < 0.05.

As depicted in Figure 4A, the average particle size of ZP was approximately 133.8 nm, suggesting that the anti-solvent precipitation method employed was effective in generating small nanoparticles. After conjugation with SSPS, the particle size of ZSP_A_-24 h and ZSP_B_-24 h in the dispersed solutions with pH 4.0 were 265.1 nm and 194.5 nm, respectively. This increase in diameter indicates the incorporation of SSPS into ZP-forming macromolecular zein-polysaccharide complexes. When the conjugation pH increased from 7.0 (ZSP_A_-24 h) to 10.0 (ZSP_B_-24 h), DG also increased, resulting in a decrease in size of the zein-based nanoparticles. This reduction is due to the fact that SSPS grafted to the protein surface provides greater spatial repulsion [41].

Moreover, the average particle size of ZSP_B_-24 h suspensions did not show a large variation from 194.5 nm to 117.2 nm in a wide pH range of 4.0–8.0, and the particle size decreased again at pH 10.0. This result implies that conjugated nanoparticles have good stability in relation to aggregation against pH (especially around the isoelectric point of zein, pI 6.2), owing to the strong spatial hindrance provided by hairy polysaccharides [42]. 

Meanwhile, the zeta-potential of the ZP was strongly cationic at around + 28.7 mV (Figure 4B). This effect can be attributed to the pH of the solution (pH 4.0) being much lower than the isoelectric point of zein. As expected, the zeta-potentials of ZSP_A_-24 h and ZSP_B_-24 h became increasingly less positive and presented negative values. These results are consistent with that of Lin et al. [43]. In the pH range of 4.0–10.0, the potential of ZSP_B_-24 h decreased from −6.25 to −37.4 mV. The main reason for this decrease could be that the covalent conjugation with the anionic polysaccharide consumed the amino group in the corn protein, and thus the nanocomplexes carried more negative electrical charge [44].

Figure 4C shows the appearance and water dispersibility of native ZP and ZSP with different pH values after 48 h. The native zein particles were fully soluble at pH 4.0 due to surface repulsion (electrostatic stabilization). Meanwhile, particles showed obvious aggregation and stratification at pH 6.0, which reduced the electrostatic repulsion and steric hindrance [45]. All the Zein/SSPS conjugated particles dissolved in water with uniform and transparent light white dispersions, which reveals that the grafting of hydrophilic polysaccharide groups on the surface of zein improved the physical stability and water solubility at different pHs. This result suggests that zein-based conjugated particles are highly stable against coalescence, and ZSP_B_-24 h appears to have better stability at pH 6.0 in particular.

#### 3.3.2. Nanoparticle Stability at pH 6.0

**Figure 5:**
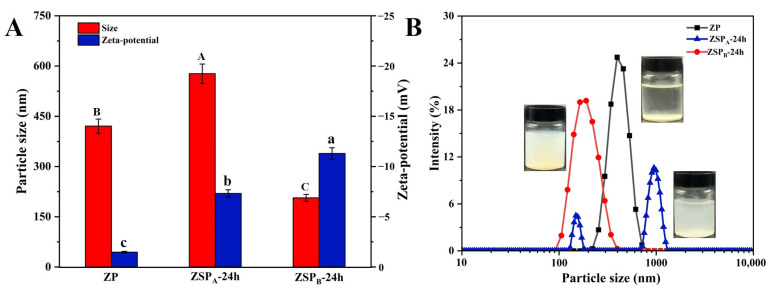
(**A**) Particle size and zeta-potential and (**B**) size distribution graph of ZP, ZSP_A_ and ZSP_B_ dispersions at pH 6.0. Different letters (A, B, C, a, b, c) indicate significant differences at *p* < 0.05.

Figure 5 illustrates the particle size, zeta-potential and size distribution of ZP and ZSP. In Figure 5B, pure ZP suspended in Milli-Q water adjusted to pH 6.0 with 1 M NaOH were found to undergo aggregation and sedimentation, which may be due to the decrease of the hydrophobic interaction and electrostatic repulsion among ZP [46]. The particle size of ZP is about 420 nm (Figure 5A), which may be the result of the supernatant of the sample being taken during particle-sizing measurement. Meanwhile, the mean particle diameter of ZSP_A_-24 h was observed to be about 560 nm and showed bimodal distributions. It might be explained by two reasons. Firstly, the insufficient coverage of the zein surface by the polysaccharide led to larger particles due to weaker steric stabilization. Secondly, the presence of a small amount of unreacted SSPS in the solution during the anti-solvent process also contributed to an increase in the average particle size [47]. However, the average particle size and zeta-potential of conjugated nanoparticles obtained through 24 h of incubation at pH 10.0 (ZSP_B_-24 h) were 206.6 nm and −11.3 mV, respectively. Compared to ZSP_A_-24 h, the average particle size and zeta-potential of ZSP_B_-24 h obviously decreased, which was caused by the increased glycation extent.

#### 3.3.3. Analysis of Nanoparticle Size and Potential under Different Reaction Conditions

**Figure 6:**
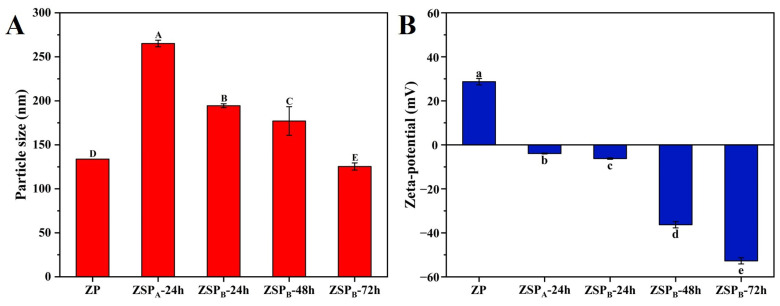
(**A**) Particle size, (**B**) zeta-potential of ZP, ZSP_A_ and ZSP_B_ dispersions at pH 6.0. Different letters (A, B, C, D, E, a, b, c, d, e) indicate significant differences at *p* < 0.05.

The influence of covalent grafting of polysaccharides to zein on its nanoparticle size and charge was investigated (Figure 6A,B). Compared to native ZP and ZSP_A_, the average particle size of ZSP_B_ decreased significantly from 196.9 nm to 158.4 nm and 129.5 nm when the reaction time increased (*p* < 0.05) This is ascribed to the higher steric repulsion provided by SSPS grafted onto the surface of the protein, preventing protein aggregation [48]. Another explanation for the reduced particle size is that the conjugated polysaccharide branching increases the volume of hydrophilic domains protruding from the protein into the environment [49]. This explanation is well supported by tertiary structural changes detected in the fluorescence spectra analysis (Figure 3). In terms of the zeta-potential, data indicates that all ZSP had mean negative charges and showed dramatic decreases with conjugation time, especially after conjugation for 72 h (−52.7 mV). This could be because the quantity of ionized groups on the particle surface was decreased by the formation of the Amadori products that consumed the dissociative amidogen groups. Similar conclusions were also observed by Yan et al. [50].

### 3.4. Wettability of Native ZP and ZSP

**Figure 7:**
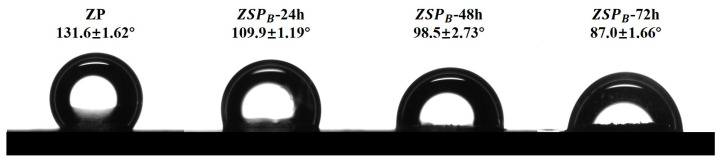
Interfacial wettability of native ZP and ZSP. Data are represented as means ± S.D (*n* = 3).

Surface wettability plays a crucial role in the ability of nanoparticles to form and stabilize Pickering emulsions. Figure 7 shows the oil-water contact angles for ZP and ZSP_B_. Pure ZP had a relatively high contact angle, 131.6°, which is consistent with previous reports [42]. This result shows that pure ZP are strongly hydrophobic and thus not suitable for stabilizing O/W emulsions. The contact angle of the ZSP_B_ sharply decreased with the extension of reaction time from 24 h to 72 h, which means that the hydrophilicity of the substrate was enhanced. The contact angle of ZSP_B_-72 h was 87°, close to 90°, which is comparable to those of the Zein/Pectin particle (84–87°) [51] and polysaccharide-protein hybrid particles from soybean (81–85°) [52], indicating that the fabricated ZSP_B_-72 h was intermediate-wetted [43] and could be a potential Pickering emulsifier.

### 3.5. Properties of Pickering Emulsions Stabilized by Native ZP and ZSP

#### 3.5.1. Visual Appearance, Optical Micrographs and Creaming Index (CI)

**Figure 8:**
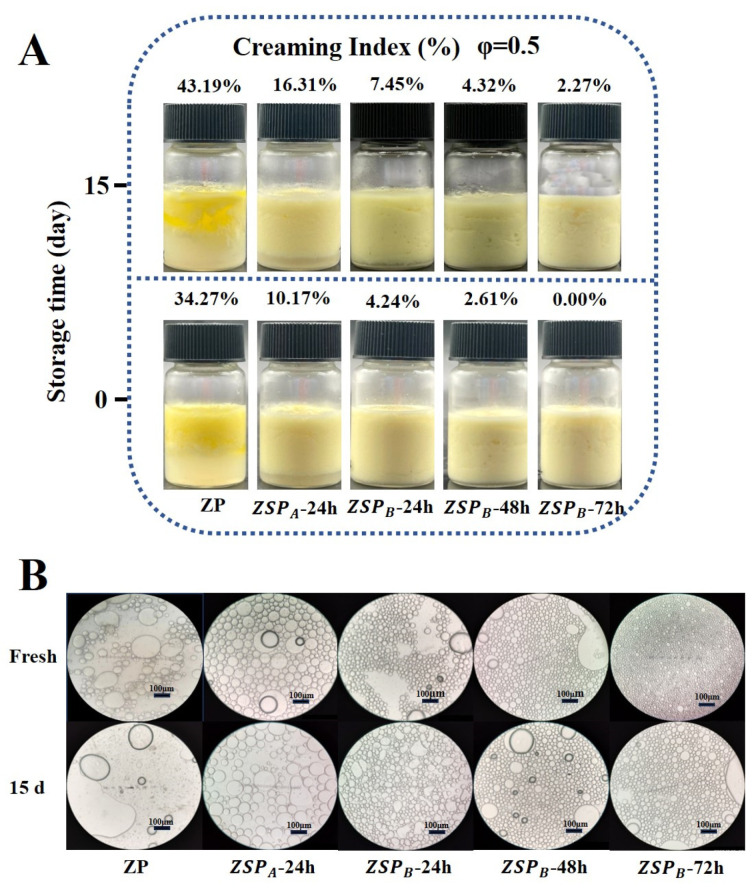
(**A**) Photograph of emulsions stabilized by conjugate particles under different storage times (0 d and 15 d), and their corresponding micrographs are shown in (**B**). The total polymer concentration of all samples was 1% (*w*/*v*), and the flaxseed oil loading was 50% (with respect to the total volume).

Stability is a crucial criterion for evaluating the shelf-life of emulsion products because the coalescence or overall phase separation of droplets could result in appearance defects [44]. As shown in Figure 8A, emulsions stabilized by native ZP immediately showed water-oil phase separation in the freshly prepared sample, whilst the ZSP_A_-24 h and ZSP_B_-24 h samples exhibited rough texture and partial phase separation. The ZSP_A_-48 h and ZSP_B_-72 h samples showed excellent emulsion stability over 15 days, probably due to the higher degree of grafting.

Further, the optical photograph was used to measure the emulsions stabilized by conjugate particles (Figure 8B). Optical microscopy observations revealed that emulsions stabilized by native ZP were largely uneven and sparsely distributed. With increasing grafting degree, the droplet size of ZSP stabilized emulsions gradually decreased, and particle size distribution was more uniform, emphasizing the improved emulsification performance through conjugation. Compared to ZP, smaller and more flexible ZSP_B_ can effectively be adsorbed on the flaxseed oil-water interface and form a dense interfacial layer, thereby preventing the accumulation of oil droplets. Moreover, the CI% values of the emulsions stabilized by ZSP_B_-72 h were significantly lower after storage for 15 days. This phenomenon also indicates that ZSP_B_-72 h has better emulsifying capacity, which is in agreement with the contact angle measurements, the values of which are closest to 90°. Previously, a Pickering lotion prepared by pea protein isolate-glucose conjugated nanoparticles also obtained similar results [53].

#### 3.5.2. Confocal Laser Scanning Microscopy (CLSM)

**Figure 9:**
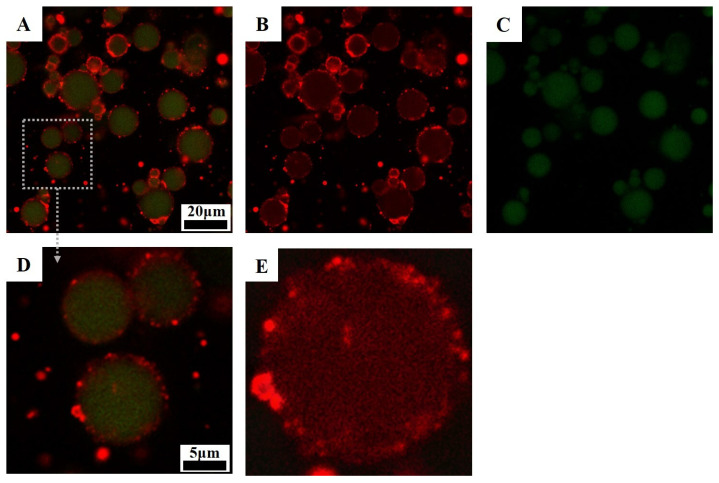
(**A**) CLSM images of the Pickering emulsions stabilized by ZSP_B_-72 h with the overlap images of (**B**,**C**); (**B**) Zein/SSPS-conjugated nanoparticles with Nile blue staining; (**C**) oil phase with Nile red staining; (**D**) an enlarged view of a part of (**A**); (**E**) an enlarged image of ZSP_B_-72 h (colored in red) adsorbed on oil droplets.

Figure 9 shows the CLSM micrographs of the ZSP_B_-72 h stabilized Pickering emulsion stored at 45 °C after 15 days. We observed a red granular fluorescence response in the emulsions, indicating the presence of zein-conjugated nanoparticles, while droplets of oil stained with Nile Red (green fluorescence) were dispersed into an aqueous phase. Moreover, the conjugation nanoparticle was tightly anchored to the oil droplet surface, resulting in the formation of a robust and stable multilayer interface film. Masoumi et al. [14] found that the accumulation of sodium caseinate-ascorbic acid conjugated nanoparticles at the oil-water interface led to the development of a viscoelastic barrier that effectively hindered flocculation and coalescence.

#### 3.5.3. Oxidative Stability

**Figure 10:**
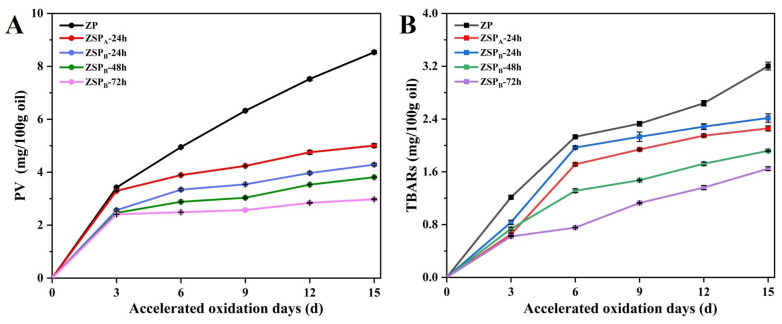
(**A**) PV and (**B**) TBARS of the Pickering emulsions stabilized by zein nanoparticles (ZP) and zein/SSPS conjugates nanoparticles (ZSP) when stored for 15 days at 45 °C.

Flaxseed oil, as the richest source of ω-3 fatty acids (50–60%), is extremely susceptible to oxidation in the presence of oxygen, metal ions and high temperatures during storage [54]. Figure 10 presents the PV and TBARS of the Pickering emulsions stabilized by ZP and ZSP when stored for 15 days at 45 °C. Compared to the emulsion stabilized by ZP, both the PV and TBARS of the emulsions stabilized by the ZSP decreased progressively. This suggests that ZSP, as an emulsion stabilizer, inhibited the oxidation of flaxseed oil in the emulsion. Moreover, there was a gradual decrease in the PV and TBARS of the emulsions stabilized by ZSP_B_ with the extension of the reaction time, which further confirmed the observation that DG was enhanced with increasing reaction time.

Previous studies have demonstrated that lipid oxidation is frequently initiated and propagated at an oil-water interface, such as with transition metals, enzymes and photosensitizers as pro-oxidants that can promote lipid oxidation [55]. The decreases in the PV and TBARS content in the Pickering emulsion loaded with flaxseed oil were mainly due to formation of a viable physical barrier, thereby delaying lipid oxidation. Furthermore, the improvement of antioxidant activity is also closely related to the abundance of the hydroxyl group or the formation of a hydrogen donor based on structural changes of zein after conjugation [56]. Accordingly, zein nanoparticles, modified by SSPS, have good antioxidant activity. Therefore, they serve as a highly effective approach for managing lipid oxidation in Pickering emulsion systems.

## 4. Conclusions

Conjugated nanoparticles, which were used as stabilizers to prepare flaxseed oil Pickering emulsions with antioxidant activity, were successfully fabricated using SSPS-grafted zein via the Maillard reaction. Analysis using various detection methods indicates that the Zein/SSPS conjugates were formed mainly by intermolecular covalent and hydrogen bonds. The resulting conjugating nanoparticles were distributed uniformly in neutral aqueous solutions compared to that of zein. Furthermore, the hydrophilicity and zeta-potential of the ZSP also increased significantly with the increase in reaction time. Pickering emulsions stabilized by ZSP_B_-72 h exhibited effective physical stability, which was validated by CLSM. Additionally, upon comparing the PV and TBARS of the emulsions stabilized by ZSP and the emulsion stabilized by ZP, the former values were significantly lower than that of the latter. The results of this study demonstrate that zein-based nanoparticles conjugated with soybean polysaccharide are promising food-grade Pickering emulsifiers.

## Figures and Tables

**Table 1:** Nomenclature and conjugation conditions of zein-based nanoparticles.

Sample Nomenclature	Ratio of Zein to SSPS (*w*/*w*)	Maillard Reaction pH	Maillard Reaction Time
ZP	1:0	--	--
ZSP_A_-24 h	1:1	pH 7.0	24 h
ZSP_B_-24 h	1:1	pH 10.0	24 h
ZSP_B_-48 h	1:1	pH 10.0	48 h
ZSP_B_-72 h	1:1	pH 10.0	72 h

## Data Availability

Data can be requested via the corresponding author.
